# Role of TREM1-DAP12 in Renal Inflammation during Obstructive Nephropathy

**DOI:** 10.1371/journal.pone.0082498

**Published:** 2013-12-16

**Authors:** Alessandra Tammaro, Ingrid Stroo, Elena Rampanelli, Froilan Blank, Loes M. Butter, Nike Claessen, Toshiyuki Takai, Marco Colonna, Jaklien C. Leemans, Sandrine Florquin, Mark C. Dessing

**Affiliations:** 1 Department of Pathology, Academic Medical Center, University of Amsterdam, Amsterdam, The Netherlands; 2 Department of Experimental Immunology, Institute of Development, Aging and Cancer, Tohoku University, Sendai, Japan; 3 Department of Pathology and Immunology, Washington University School of Medicine, St. Louis, Missouri, United States of America; 4 Department of Pathology, Radboud University Nijmegen Medical Center, Nijmegen, The Netherlands; Universtiy of Maryland Schoool of Medicine, United States of America

## Abstract

Tubulo-interstitial damage is a common finding in the chronically diseased kidney and is characterized by ongoing inflammation and fibrosis leading to renal dysfunction and end-stage renal disease. Upon kidney injury, endogenous ligands can be released which are recognized by innate immune sensors to alarm innate immune system. A new family of innate sensors is the family of TREM (triggering receptor expressed on myeloid cell). TREM1 is an activating receptor and requires association with transmembrane adapter molecule DAP12 (DNAX-associated protein 12) for cell signaling. TREM1-DAP12 pathway has a cross-talk with intracellular signaling pathways of several Toll-like receptors (TLRs) and is able to amplify TLR signaling and thereby contributes to the magnitude of inflammation. So far, several studies have shown that TLRs play a role in obstructive nephropathy but the contribution of TREM1-DAP12 herein is unknown. Therefore, we studied TREM1 expression in human and murine progressive renal diseases and further investigated the role for TREM1-DAP12 by subjecting wild-type (WT), TREM1/3 double KO and DAP12 KO mice to murine unilateral ureter obstruction (UUO) model. In patients with hydronephrosis, TREM1 positive cells were observed in renal tissue. We showed that in kidneys from WT mice, DAP12 mRNA and TREM1 mRNA and protein levels were elevated upon UUO. Compared to WT mice, DAP12 KO mice displayed less renal MCP-1, KC and TGF-β1 levels and less influx of macrophages during progression of UUO, whereas TREM1/3 double KO mice displayed less renal MCP-1 level. Renal fibrosis was comparable in WT, TREM1/3 double KO and DAP12 KO mice. We conclude that DAP12, partly through TREM1/3, is involved in renal inflammation during progression of UUO.

## Introduction

Fibroproliferative diseases, including chronically kidney disease, are a leading cause of morbidity and mortality and can affect all tissues and organ systems [1]. Tubulo-interstitial damage is a common finding in the chronically diseased kidney whereby the degree of tubulo-interstitial damage is a major determinant of renal function and outcome. Tubulo-interstitial injury is characterized by infiltration of leukocytes and myofibroblast accumulation with excessive deposition of extracellular matrix (ECM) [2]–[5]. As inflammation and interstitial fibrosis progress, tubular atrophy occurs which leads to renal dysfunction and finally to end-stage renal disease. Because the extent of fibrosis is an important predictor for outcome, much research has been put into the mechanisms behind this phenomenon. Unilateral ureteral obstruction (UUO) is a model frequently used in mice that mimics features observed in tubulo-interstitial injury including influx of leukocytes and accumulation of (myo)fibroblast followed by ECM deposition and tissue loss 2–5. During tissue injury, like in UUO, damaged cells are able to elicit danger signals so called ‘danger-associated molecular patterns’ (DAMPs). These DAMPs are endogenous ligands which are able to alert the innate immune system through recognition by pattern-recognition receptors (PRR) including Toll-like receptors (TLRs) [6], [7]. Recently, a new family of receptors were discovered which share intracellular signaling cascade of TLRs called TREM (triggering receptor expressed on myeloid cell). TREM1 is an activating receptor expressed on neutrophils, monocytes and some types of macrophages [8], [9]. TREM1 lacks an intracellular signaling motif and associates with its adaptor molecule TYROBP (commonly called DAP12; DNAX-activating protein-12) to induce cytokine production. No specific ligand for TREM1 has been found so far but HMGB1, known to be increased following UUO, is a potential candidate [10]–[13]. Besides its own direct intracellular signaling cascade pathway, TREM1-DAP12 activation has a cross-talk with intracellular signaling pathways of several TLRs and is able to amplify TLR signaling and can thereby contribute to the magnitude of inflammation. So far, several studies have shown that TLR2, TLR4 and their intracellular adopter molecule MyD88 play part in mechanisms of renal fibrosis [11], [12], [14]–[16]. We hypothesis that TREM1-DAP12 signaling contributes to the magnitude of inflammation during UUO. To our knowledge, no studies about TREM1-DAP12 in renal injury or fibrosis are known. We investigated localization of TREM1 in human kidneys suffering from obstructive nephropathy. Subsequently, we investigated the contribution of TREM1-DAP12 in renal fibrosis by subjecting wild-type, TREM1/3 KO and DAP12 KO mice to UUO model.

## Materials and Methods

### Patients

Kidney samples were obtained from patients diagnosed with terminal hydronephrosis characterized by extensive fibrosis and tubular atrophy at the Academic Medical Center as described before [11]. As control, we used a protocolar biopsy from a renal transplant patient with stable graft function, obtained 6 months after transplantation obtained from a cohort study at Academic Medical Center as described [17]. All renal biopsies were taken for diagnostic purposes only. This research project used only left-over biological material, anonyms and delinked from patient records, and as such was not subjected to any requirement for ethical review or approval.

### Mice

Pathogen-free 9 to 12-week old female WT C57Bl/6 mice were purchased from Charles River Laboratories. TREM1/3 double KO mice were generated as recently described before [18]. TREM1/3 double KO mice were generated because TREM1 and TREM3 are highly homologous and likely to be redundant. Because TREM3 is a pseudogene in humans, TREM1/3 double KO mouse would more closely mimic human TREM1 deficiency. TREM1/3 double KO mice (further referred to as TREM1/3 KO mice) were fully backcrossed to a C57Bl/6 background. DAP12 KO mice backcrossed to C57bl/6 background were generated as before [19]. TREM1/3 KO and DAP12 KO mice were bred in the animal facility of the Academic Medical Center in Amsterdam, the Netherlands. Sex and age matched mice were used. The Animal Care and Use Committee of the University of Amsterdam approved all experiments.

### Unilateral Ureteral Obstruction

Progressive renal injury was induced by unilateral ureteral obstruction (UUO) as described before [11], [12], [20]–[22]. Briefly, the right ureter was ligated under 2% isoflurane-induced anesthesia. After abdomen was closed, mice received a subcutaneous injection of 50 µg/kg buprenorphin (Temgesic, Shering-Plough). Mice were sacrificed 1, 3, 7 or 14 days after surgery. (Un)obstructed kidney was divided for immunohistochemistry (paraffin) or snap-frozen in liquid nitrogen for RNA and protein determination.

### Immunohistochemistry

Human and murine renal tissue was fixed in 10% formalin and embedded in paraffin. Tissue sections of kidney (4 µm) were dried overnight and deparaffinized. Antigen retrieval, blocking of non-specific binding, incubation with primary and secondary antibody and development were performed as described before [11], [12], [20]–[22]. For TREM1 immunohistochemistry, human renal tissue section were pretreated with citrate buffer (10 mM Sodium Citrate, pH 6.0), incubated with TREM1 antibody (Rabbit-anti Human; Sigma HPA005563), followed by BrightVision Poly- HRP-Anti Rabbit (Immunoligic) and developed using 1% H_2_O_2_ and DAB (Sigma) in 0.05 M Tris-HCl (pH 7.9). On mouse renal sections, detection of macrophages (rat anti-mouse F4/80; Serotec), granulocytes (rat anti-mouse Ly6/G-Fitc; Pharmingen), α-smooth muscle actine (mouse-anti-human αSMA-IgG2a; Dako), collagen type I (rabbit polyclonal to collagen type I; Genetek) and apoptotic cells (rabbit anti-human active caspase-3; Cell Signalling Technology) was performed with appropriate secondary antibody and developed using 1% H_2_O_2_ and DAB (Sigma) in 0.05 M Tris-HCl (pH 7.9). For the quantification of macrophages, αSMA and collagen type I staining, photos of kidney tissue slides (magnification x20) were taken and analyzed using ImageJ v1.47. Percentage of positive DAP staining was related to total tissue area. Granulocytes and apoptotic cells were counted in 10 high power field (HPF, magnification x40). Histopathological scoring for Periodic Acid-Schiff Diastase (PASD) stained renal tissue sections were performed in the cortex. Criteria for measuring tubular damage were epithelial flattening, tubular dilatation and brush border loss. Sections were graded from 0 = 0%, 1 = 0–10%, 2 = 10–25%, 3 = 25–50%, 4 = 50–75% and 5 = more than 75% damaged tubules in the cortex. Interstitial edema was estimated using a 4-point scale: 0 = normal; 1 = mild; 2 = moderate; 3 = severe and 4 = extensive edema. Tubular injury and edema scoring was performed in 10 randomly chosen, non-overlapping fields (magnification x400).

### In Situ Hybridization

Fragments of the Trem1 and Tyrobp cDNAs were obtained by PCR using specific primers Trem1 F: 5′-CACAGAGGCAGTCGTTGGAG, R: 5′- ACATCTGAAGAACCCTTGGTCA; Tyrobp F: 5′- GTGGGAGGATTAAGTCCCGTA, R: 5′- CAGGGAGGTACCCTGTGGAT. The PCR product was cloned into pGEM-T-easy vector (Promega Benelux BV; Leiden, The Netherlands) and verified by sequence analysis. Digoxigenin-11-UTP-labeled riboprobes were synthesized from SphI or PstI linearized plasmids by in vitro transcription reaction with T7 or Sp6 polymerase (Roche Applied Science). After the labeling reaction, the template DNA was digested using RQ-DNase (Promega), and the labeled RNA probe was precipitated. The probe was dissolved in 10 mM Tris-HCl,1 mM EDTA pH 8 and 1U RNAsin/ul and its concentration measured by Nanodrop. The non-radioactive in situ hybridization procedure was performed, in general, as partly described before [23]. Ten-µm-thick sections were prepared and mounted onto coated slides. After deparaffinization and hydration, sections were incubated with 20 µg/ml proteinase K dissolved in PBS for 15 min at 37C. The proteinase K activity was blocked by rinsing the sections in 0.2% glycine in PBS for 5 min. After two washes in PBS for 5 min, the sections were postfixed for 10 min in 4% PFA and 0.2% glutaraldehyde in PBS, followed by two washes in PBS for 5 min. Before prehybridization, a hydrophobic barrier was drawn around each individual section using an ImmEdge pen (Vector Laboratories; Burlingame, CA). This barrier allows treatment of each individual section with a different RNA probe. After prehybridization for at least 1 hr at 70C in hybridization mix (50% formamide, 5X SSC (20X SSC; 3 M NaCl, 0.3 M tri-sodium citrate, pH 4.5), 1% blocking solution (Roche), 5 mM EDTA, 0.1% 3–[(3-Cholamidopropyl) dimethylammonio]-1-propanesulfonate (Sigma; Steinheim, Germany), 0.1 mg/ml heparin (BD Biosciences; Erembodegem, Belgium), and 1 mg/ml yeast total RNA (Roche), a digoxigenin (DIG)-labeled probe was added to the hybridization mix in a final concentration of 3 ng/µl. After overnight hybridization, the sections were rinsed with 2X SSC, followed by 2 washes with 50% formamide, 2X SSC, pH 4.5, at 65C for 30 min and 1 hour, and finally, three washes in TBS tween 0.05% at room temperature. Subsequently, the sections were incubated for 30 minutes with 2% blocking solution in PBS-T, followed by overnight incubation with 2% blocking solution in PBS-T containing 100 mU/ml anti-DIG Fab fragment covalently coupled to alkaline phosphatase (AP) (Roche). Probe binding was visualized using nitro blue tetrazolium chloride and 5-bromo-4-chloro-3-indolyl-phosphate, toluidine-salt as the chromogenic substrate for anti-DIG-AP, according to the manufacturer's protocol (Roche). After staining times of 4–6 hr at room temperature, the color development was stopped by rinsing in double-distilled water. The sections were dehydrated in a graded ethanol series, rinsed in xylene, and embedded in Pertex. DAP12 and TREM1- in situ hybridization was not observed using sense strains (data not shown).

### Isolation and stimulation of primary renal tubular epithelial cells

Primary renal tubular epithelial cells (TECs) from WT mice were cultured as described before [11], [12], [20]–[22] and cultured in HK2 medium in 6-wells plate until monolayer (DMEM/F12 supplemented with 10% FCS, Penicillin/streptomycin, L-glutamine (all Invitrogen), 1% ITS and 1% S1 hormone mixture (Sigma)). TECs were stimulated with 1 or 5 ng/ml recombinant human TGF-β1(R&D systems) for 1 or 3 days.

### RNA purification and rtPCR

Total RNA was isolated from 10–15 renal frozen tissue slides (20 µM) and *in vitro* cell culture using Trizol reagent (Invitrogen) according to manufacturer's protocol. RNA samples were quantified by spectrophotometry (Nanodrop) and converted to cDNA using oligo-dT. mRNA expression was analyzed by RT-PCR with SYBR green PCR master mix on a Roche LC480 lightcycler. Relative expression was analyzed using LinRegPCR v12.4. Gene expression was correlated to housekeeping gene (TATA-binding protein; TBP). Primer sequences: Sirpb1 forward (F): 5′-GGTGGCACTGAGTTGTTAGTCC, reverse (R): 5′-AAAGGTCACTGTCTGCTGAGG; Pilrb1 F: 5′-TGGAACCCAACTCAACGTG, R: 5′-GGATTCCTCTGGTCGCTTG; Pilrb2 F: 5′-GCACACAAGGGACAAGAGGAA, R: 5′-TGCTGGTTCTCCATCCTGACT; Cd200r3 F: 5′-TACAACCAGCATCCTGCCTTC, R: 5′-TGCTTCTCGGAATCTCAGCAC; Cd200r4 F: 5′-CACTGTGACTGTCAGGAGCAC, R: 5′-GGGGGTGGTCATTGTACCTT; Clec5a F: 5′-TGGAGAGAAAAAGTGGCGCT R: 5′-CTGGGCTGGTTTCAGTCACA; Trem1 F: 5′-GCGTCCCATCCTTATTACCA, R: 5′-AAACCAGGCTCTTGCTGAGA; Trem2 F: 5′-ACCCACCTCCATTCTTCTCC, R: 5′-GGGTCCAGTGAGGATCTGAA; Trem3 F: 5′-AAAGCTGGGCCTAAGGTGTT, R: 5′-CCCAGATTGTCCCCAATACA; Cd300lb F: 5′-GACACGGACACTTACTGGTGT, R: 5′-GCACCAACCAAGATGAGCAA; AF251705 F: 5′-GACCCAGTCACAGGTTCGAG, R: 5′-AGAAGTAGAGGCTGCTCCGA; Siglech F: 5′-CCATGTCTCTGGAAGAGTTGGT, R: 5′-GTATGGGATCTCTTGCTGAGGA; Siglec15 F: 5′-AGGCCAGCGTCTACCTGTTC, R: 5′-TGGTGATGGCTGAGGAGTTC; Tyrobp F: 5′-CGAAAACAACACATTGCTGA, R: 5′-GGGCATAGAGTGGGCTCAT; Treml-1 F: 5′ -AGAATCGGAACCGGAAGTCT, R: 5′ -GGCCATCACAGCAAATATCA; Treml-2 F: 5′-CACCTGTGGTGTTGGTCGTA, R: 5′-CCTTCTGAACCCACTGGAAA; Treml-4 F: 5′-ATGGACTCCTCCTGCTCAAG, R: 5′-AGATGTGGCTAACCGTGTCC, Tlr2 F: 5′-GGGGCTTCACTTCTCTGCTT, R: 5′-AGCATCCTCTGAGATTTGACG, Tlr4 F: 5′ -GGACTCTGATCATGGCACTG, R: 5′-CTGATCCATGCATTGGTAGGT and TBP F: 5′-GGAGAATCATGGACCAGAACA, R: 5′-GATGGGAATTCCAGGAGTCA.

### ELISA and Western Blot

For ELISA, snap-frozen kidney part was homogenized in Greenberger Lysis buffer (300 mM NaCl, 15 mM Tris, 2 mM MgCl, 1 mM CaCl_2_ and 1% Triton X100, pH 7.4 with 100 µg/ml pepstatin A, leupeptin and aprotinin mix), incubated for 30 minutes at 4°C, centrifuged at 1500 g and stored in −80°C. Levels of keratineocyte-derived cytokine (KC), monocyte chemotactic protein (MCP)-1 and transforming growth factor (TGF)-β1 (all R&D Systems) were measured in kidney homogenate according to manufacturer's instructions. Protein levels were corrected for total protein level present in sample using BioRad protein assay (BioRad Laboratories) with IgG as standard. For western blot, kidney lysates were prepared from 15 frozen sections (30 µm thick) or from primary TEC in culture, incubated at 4°C for 30 min in RIPA buffer containing 50 mM Tris pH7.5, 0.15 M NaCl, 2 mM EDTA, 1% deoxycholic acid, 1% NP-40, 4 mM sodium orthovanadate, 10 mM sodium fluoride, 1% protease inhibitor cocktail (P8340, Sigma). The lysates were then centrifuged at 12000 rpm for 15 minutes and the supernatants were collected and stored at −20°C. SDS-polyacrylamide gel electrophoresis was carried out in 10% gradient slab gels and proteins were electrophoretically transferred onto methanol-activated polyvinylidene fluoride (PVDF) microporous membranes (Millipore, Etten Leur, The Netherlands). Membranes were blocked for one hour with 5% bovine serum albumin (Sigma) in Tris-buffered saline containing 0.1% Tween 20 (TBS-T), followed by overnight incubation at 4°C with primary rabbit anti-mouse/human TREM1 (Abcam ab104413, Cambridge, UK). HRP-conjugated secondary antibodies (DAKO, Glostrup, Denmark) were incubated for one hour at room temperature, and HRP activity was visualized with ECL-reagent (Amersham Pharmacia Biotech, Roosendaal, The Netherlands. β-actin was used as loading control and detected by mouse IgG1 anti-β-actin (Abcam, Cambridge, UK). Densitometric quantification analysis was performed on imagines of scanned films using the image processing program ImageJ (National Institute of Health, US).

### Statistical analysis

Comparison between >2 groups was analyzed using Kruskal-Wallis test followed by Dunn's Multiple Comparison test. Level of significance between 2 groups was determined by Mann-Whitney. Data are expressed as mean ± standard deviation (SD). Eight mice per group were analyzed at every time point. P<0.05 was considered statistically significant.

## Results

### TREM1 expressing cells in hydronephrosis

**Figure 1:**
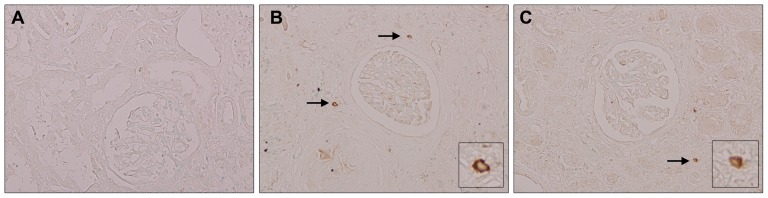
TREM1 expression and localization during hydronephrosis. TREM1 expression on renal biopsies from renal transplant patients with stable graft function (A) and from patients with obstructive hydronephrosis (B+C). Arrow indicates examples of TREM1-positive cells. Inserts display enlargement of TREM1-positive cells. Magnification x20.

Renal fibrosis is characterized by accumulation of interstitial leukocytes and myofibroblasts, induction of inflammation with eventual tubular atrophy and loss of renal function. Fibrosis and tubular atrophy are characteristic features of hydronephrosis. TREM1 expression has never been reported in human kidney disease and could contribute in inflammatory processes during hydronephrosis. In renal tissue from patients with hydronephrosis, TREM1 was expressed on tubulointerstitial cells (figure 1) which was not detectable in renal biopsies from renal transplant patients with stable graft.

### TREM1-DAP12 expression upon murine UUO

**Figure 2:**
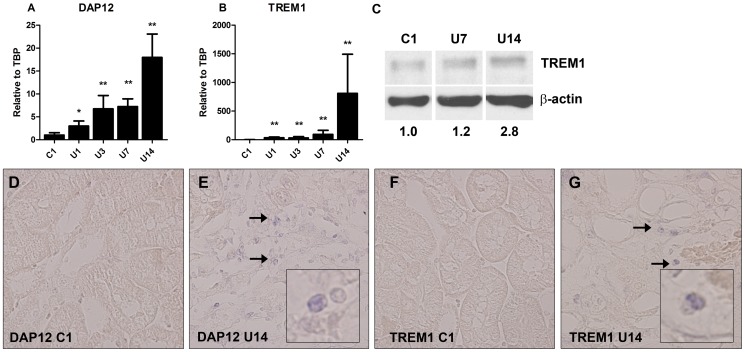
Renal levels of DAP12 and TREM1 upon UUO. Frozen kidney sections were cut and dissolved in TRIzol (A+B) or RIPA buffer (C) to isolate RNA and protein respectively. Renal mRNA levels of DAP12 (A) and TREM1 (B) were determined in WT mice, 1, 3, 7 and 14 days following UUO. Contralateral unobstructed kidney 1 day after UUO was used as control (C1). Gene expression is relatively expressed to housekeeping gene TBP and control is adjusted to relative expression of 1. (C): representative TREM1 western blot (WB) of kidney tissue in control WT mice (C1), 7 (U7) and 14 (U14) days after UUO. Ten µg of total protein was loaded in each lane. Values below WB indicate TREM1 protein densitometric quantification normalized to loading control β-actin and control (C1) was set to 1. *P<0.001, **P<0.0005 vs. control. DAP12 (D+E) and TREM1 (F+G) in situ hybridization was performed on renal paraffin section from WT mice 14 days following UUO. Contralateral unobstructed kidney 1 day after UUO was used as control. Arrows indicate example of positive cells. Inserts display enlargement of positive cells. Magnification x40.

Murine UUO is a frequently used model which represents progressive renal injury and fibrosis and has the same pathology as hydronephrosis. We screened for DAP12 and TREM1 mRNA levels in kidneys from WT mice following UUO. DAP12 and especially TREM1 mRNA levels significantly increased following UUO with highest expression at day 14 (DAP12: 18-fold and TREM1: 800-fold compared to control; figure 2). In situ hybridization showed that DAP12- and TREM1-mRNA positive tubulointerstitial cells are present upon UUO which was not present in control kidneys. Following UUO, TREM1 expression also increased on protein level, especially 14 days after UUO (figure 2C). TREM1 expression was not observed in kidneys from TREM1/3 KO mice (data not shown). Increased renal TREM1 expression could originate from infiltrating TREM1-expressing myeloid cells or, although yet unknown, upregulation of TREM1 on renal cells like TECs. *In vitro* studies showed that DAP12 and TREM1 mRNA level in TECs were low and remained similar upon stimulation with 1 or 5 ng/ml TGF-β1for 1 or 3 days (data not shown). In addition, we showed TREM1 expression on protein level on TECs which remained similar upon stimulation with 1 or 5 ng/ml TGF-β1for 3 days (figure S1).

### TREM (like) family and TLR expression in WT, TREM1/3 KO and DAP12 KO mice

**Figure 3:**
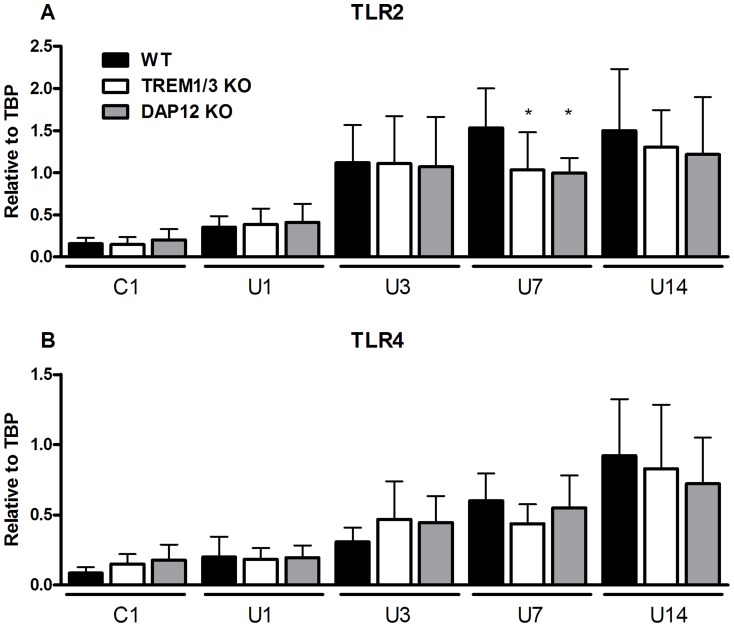
TLR2 and TLR4 expression upon UUO in WT, TREM1/3 KO and DAP12 KO mice. Frozen kidney sections were cut and dissolved in TRIzol to isolate RNA. Renal mRNA levels of TLR2 (A) and TLR4 (B) were determined in WT (black bars), TREM1/3 KO (white bars) and DAP12 KO (grey bars) 1, 3, 7 and 14 days following UUO. Contralateral unobstructed kidney 1 day after UUO was used as control (C1). Gene expression is relatively expressed to housekeeping gene TBP and control is adjusted to relative expression of 1. *P<0.05, **P<0.01 versus WT at indicated time point.

The murine family of TREM receptors includes Trem1, Trem2, Trem3 and Trem-like transcript (Treml) -1, -2 and -4. We screened these genes in TREM1/3 KO mice (and DAP12 KO mice) to exclude any potential compensation of these genes due to mutation in KO mice. Renal mRNA levels of TREM2, Trem-like transcript (Treml) -1, -2 and -4 were similar between WT, TREM1/3 KO and DAP12 KO mice in control kidneys (data not shown). TREM1-DAP12 pathway is involved in modulating the TLR-signaling cascade, most likely TLR2 and TLR4 [24]. We next investigated TLR2 and TLR4 expression in WT, TREM1/3 KO and DAP12 KO mice following UUO. In WT mice following UUO, renal TLR2 mRNA (day 3, 7 and 14) and TLR4 mRNA (day 7 and 14) increased significantly compared to control as described earlier [11] [12], (figure 3, statistics not displayed). Renal TLR2 and TLR4 mRNA increased similar in all three groups following UUO except for reduced TLR2 mRNA levels in TREM1/3KO and DAP12 KO mice, 7 days after UUO (figure 3).

### Inflammation in WT, TREM1/3 KO and DAP12 KO mice upon UUO

**Figure 4:**
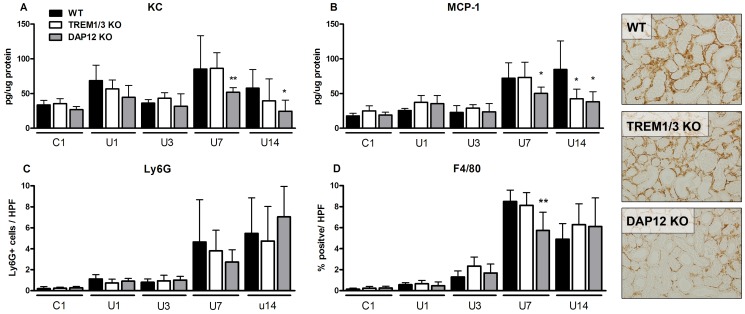
Inflammation upon UUO in WT, TREM1/3 KO and DAP12 KO mice. Renal tissue was processed to determine KC (A) and MCP1 (B) expression, granulocyte (C) and macrophage (D) influx in WT (black bars), TREM1/3 KO (white bars) and DAP12 KO (grey bars) mice 1, 3, 7 and 14 days following UUO. Contralateral unobstructed kidney 1 day after UUO was used as control (C1). Ly6G cells were counted per high power field (HPF, magnification x400). F4/80 staining is expressed as % positive staining per HPF, magnification x20. *P<0.05, **P<0.01 versus WT at indicated time point. Pictures are representative photographs of F4/80 IHC staining of kidney tissue slides from WT, TREM1/3 KO and DAP12 KO mice, 7 days following UUO (magnification x20).

Typically, progressive renal injury is characterized by production of chemokines and recruitment of inflammatory cells including macrophages and granulocytes [11] [12]. In WT mice following UUO renal KC (day 1 and 3) and MCP1, Ly6G staining and F4/80 staining (all day 7 and 14) increased significantly compared to their control (figure 4; statistics not displayed). Compared to WT mice, DAP12 KO mice displayed less renal KC and MCP-1 levels (figure 4A+B, day 7 and 14) and reduced level of macrophages 7 days after UUO (figure 4D). TREM1/3 KO mice displayed less renal MCP-1 levels 14 days after UUO (figure 4B). The number of infiltrating granulocytes progressively increased upon UUO over time and displayed similar pattern in WT, TREM1/3 and DAP12 KO mice (figure 4C).

### Fibrosis marker upon UUO

**Figure 5:**
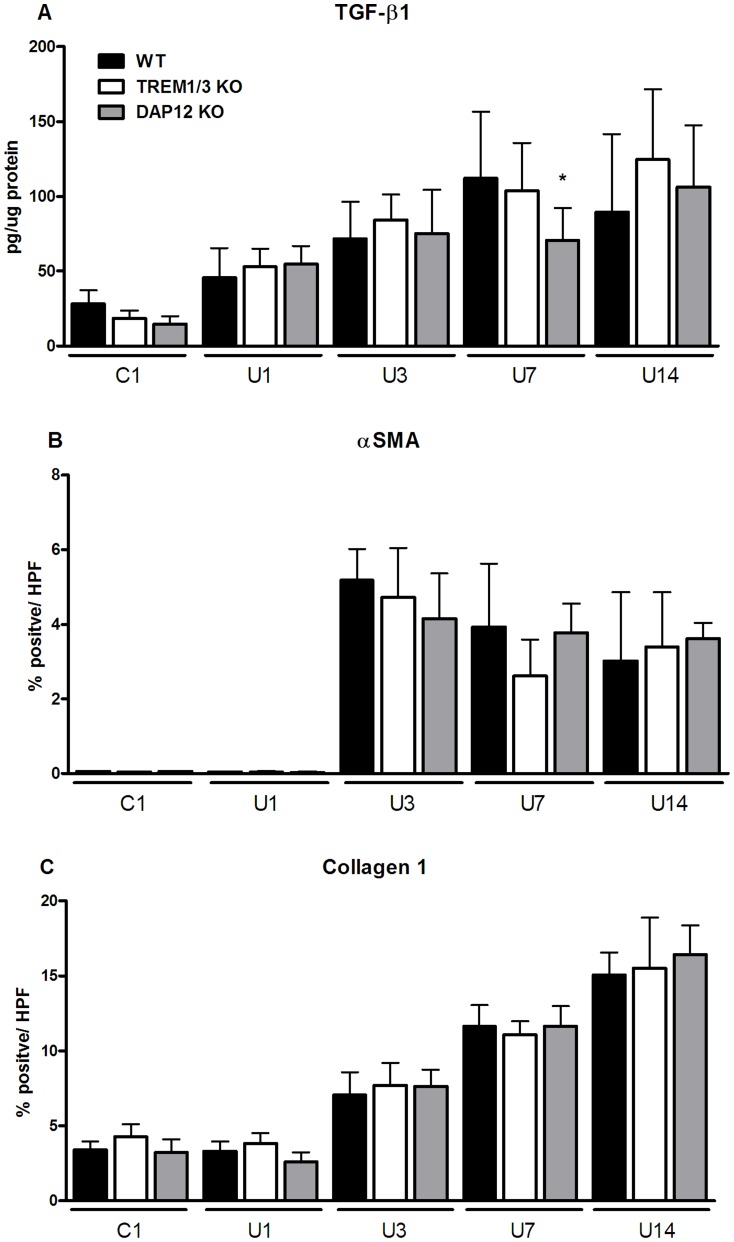
Renal fibrosis markers upon UUO. Renal tissue was processed to determine TGF-β1expression in kidney homogenate (A) and αSMA (B) and collagen type I (B) staining on kidney tissue slides in WT (black bars), TREM1/3 KO (white bars) and DAP12 KO (grey bars) 1, 3, 7 and 14 days following UUO. TGF-β1is corrected for total protein level per sample. αSMA and collagen type I staining is expressed as % positive staining per high power field. Contralateral unobstructed kidney 1 day after UUO was used as control (C1). *P<0.05 versus WT at indicated time point.

To establish the effect of TREM1/3 and DAP12 deficiency on the development of fibrosis we measured renal TGFβ, accumulation of αSMA expressing cells and collagen type I deposition. In WT mice following UUO renal TGF-β1, αSMA staining and Collagen 1 staining (all day 3, 7 and 14) increased significantly compared to control (figure 5; statistics not displayed). Compared to WT mice, DAP12 KO mice displayed less renal TGF-β1 levels, 7 days after UUO (figure 5A). The accumulation of αSMA expressing cells and collagen type I deposition increased similar in WT, TREM1/3 KO and DAP12 KO mice (figure 5B+C).

### Tubular injury and interstitial edema upon UUO

**Figure 6:**
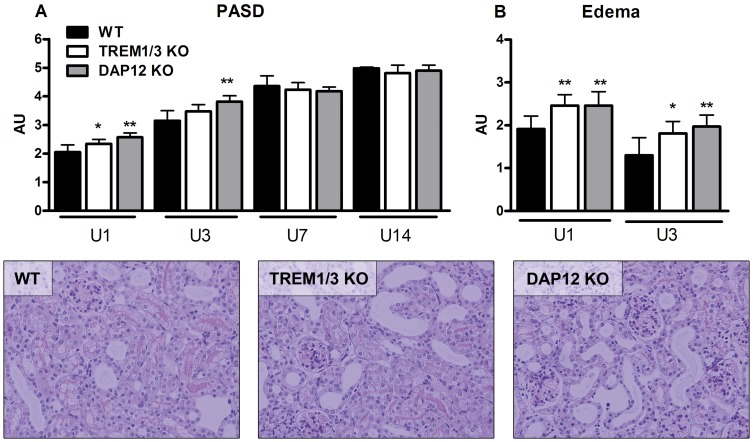
Tubular injury and edema upon UUO. Kidney tissue slides were cut and quantified for PASD (A) and edema score (B) in WT (black bars), TREM1/3 KO (white bars) and DAP12 KO (grey bars) mice following UUO. PASD score was graded according to % of damaged tubuli on an increasing 5-point scale (see methods section). Interstitial edema score was graded according to severity on an increasing 4-point scale (see methods section). *P<0.05, **P<0.01 versus WT at indicated time point. Pictures are representative photographs of PASD staining of kidney tissue slides from WT, TREM1/3 KO and DAP12 KO mice, 3 days following UUO (magnification x20).

We counted apoptotic TECs and scored tubular damage and edema on renal tissue sections. Presence of caspase3+ TECs was observed especially 7 and 14 days after UUO and numbers were not significantly different between groups (data not shown). In WT mice, tubular injury (as depicted by PASD score) significantly increased 3, 7 and 14 days after UUO compared to day 1 (figure 6; statistics not displayed). A small but significant increase in tubular damage was observed in TREM1/3 KO (day 1) and DAP12 KO mice (day 1+3) compared to WT mice which was in line with enhanced interstitial edema in these mice (figure 6).

### Other DAP12-associated receptors

**Figure 7:**
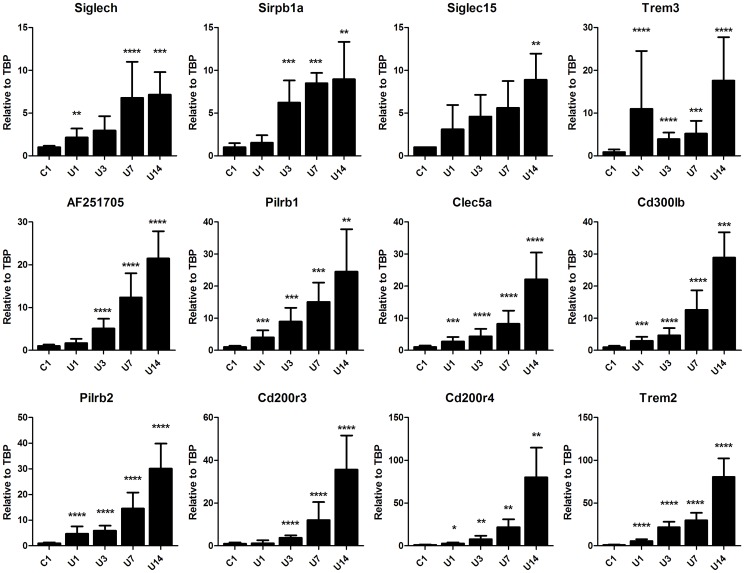
Renal mRNA levels of DAP12-associated myeloid receptors upon UUO. Frozen kidney sections were cut and dissolved in TRIzol to isolate RNA. Renal mRNA levels of Siglech, Sirpb, Siglec15, Trem3, AF251705, Pilrb1, Clec5a, Cd300lb, Pilrb2, Cd200r3, Cd200r4 and Trem2 were determined in WT mice, 1, 3, 7 and 14 days following UUO. Contralateral unobstructed kidney 1 day after UUO was used as control (C1). Genes are presented top-left to bottom-right in order of magnitude (expression on day 14 compared to control). Gene expression is relatively expressed to housekeeping gene TBP and group C1 is adjusted to relative value of 1. *P<0.01, **P<0.005, ***P<0.001, ****P<0.0005 vs. control.

TREM1/3 KO mice displayed only partly similar phenotype compared to DAP12 KO mice. Possibly, also other DAP12-associated receptors contribute to the induction of inflammation upon UUO. We screened for other DAP12-associated myeloid receptors including Sirpb1 (signal-regulatory protein beta 1A), Pilrb1+2 (paired immunoglobin-like type 2 receptor beta), Cd200r3, CD200r4, Clec5a (C-type lectin domain family 5, member a), Trem2, Trem3, Cd300lb (CD300 antigen like family member B), AF251705 (cDNA sequence AF251705 known as MAIR-II), Siglech (sialic acid binding Ig-like lectin H) and Siglec15[25]. Renal mRNA levels of mentioned DAP12-associated myeloid receptors all gradually increased upon UUO with highest expression at day 14 (figure 7). In order of magnitude, 14 days after UUO renal mRNA levels of Siglech (7.2-fold), Sirpb (8,1-fold), Siglec15 (8,9-fold), Trem3 (16,1-fold), AF251705 (21,5-fold), Pilrb1 (21,6-fold), Clec5a (22,2-fold), Cd300lb (28,9-fold), Pilrb2 (30,1-fold), Cd200r3 (35,7-fold), Cd200r4 (80,1-fold) and Trem2 (80,6-fold) were significantly increased compared to control (P<0.005 to P<0.0005 vs. control). It remains to be investigated which, next to TREM1, DAP12-associated receptors contributes to inflammation upon UUO.

## Discussion

Tubulo-interstitial damage is characterized by inflammation and myofibroblast accumulation with excessive deposition of extracellular matrix (ECM) [2]–[5]. Inflammatory response can be triggered by TREM1-DAP12 pathway activation through amplification of TLR-signaling. Whereas the contribution of TLRs in obstructive nephropathy is known, the contribution of TREM1-DAP12 herein is not. We showed that TREM1-expressing cells infiltrate kidney tissue from patients suffering obstructive nephropathy. In mice, DAP12 and especially TREM1 expression increased during progression of UUO. DAP12, partly through TREM1/3, contributes to inflammation during progression of UUO but does not affect fibrosis.

TREM1 expression has been reported in several human myeloid and non-myeloid cells and in inflamed human lung, skin and intestinal tissue [26]–[30] and is expressed to a lesser extend on lymphocytes [28]. To our knowledge we are the first to report TREM1 expression in kidney disease. These TREM1 positive cells could contribute to the magnitude of inflammation and TREM1-DAP12 signaling and was therefore further investigated using TREM1/3 KO and DAP12 KO mice. TREM1 KO and TREM1/3 KO mice were described only very recently [18] and so far, studies on TREM1 signaling were generally performed using agonistic or inhibitory antibodies and/or peptides. TREM1-DAP12 signaling was shown to be important in modulating the innate immune response, most likely by amplifying TLR-induced inflammation. TREM1-DAP12 is known to be involved in magnitude of inflammation during sepsis and bacterial infections [9] [31]. However, the contribution of TREM1-DAP12 during tissue injury is limited. Inhibition of TREM1 reduced inflammatory symptoms and improved survival during mesenteric ischemia-reperfusion injury and hemorrhagic shock [32] [33]. Similar, pretreatment with TREM1 inhibitor improved the hepatic and renal dysfunction in experimental model of severe acute pancreatitis [34]. A study by Lech *et al.* reported enhanced TREM1 and DAP12 mRNA levels following renal IR and UUO but so far, no studies about the contribution of TREM1-DAP12 signaling in kidney diseases are known [35]. We showed that DAP12 mRNA and TREM1 mRNA/protein levels are elevated 14 days after induction of UUO (figure 2). We believe that increased levels most likely originate from infiltration and activation of TREM1-DAP12 expressing cells like granulocytes and macrophages [36] and not from increased expression on renal cells like TECs. So far, we were unsuccessful in localizing TREM1 or DAP12 positive cells on murine renal tissue sections using immunohistochemistry with commercially available antibodies (data not shown). Although a specific ligand for TREM1 is unknown, endogenous DAMPs like HSP70 and HMGB1 have been suggested [10]. Of these two candidates, HMGB1, but not HSP70, is known to be upregulated during progression of UUO [11], [12], [37]. HMGB1 is also a well known TLR ligand and it remains to be investigated if HMGB1 signals through TREM1/3 and/or TLR during UUO.

During later phase of UUO, TREM1/3 KO and especially DAP12 KO mice displayed reduced levels of renal chemokines compared to WT mice showing that DAP12 signaling, partly through TREM1/3, contributes to renal inflammation upon progression of UUO (figure 4). The delayed macrophage influx in DAP12 KO mice could be related to lower renal levels of MCP1. Otherwise, delayed macrophage influx could be related to intrinsic feature of DAP12 deficient macrophages; DAP12-deficient macrophages are known to have a reduced migration capacity towards MCP1 [38]. But at day 14, macrophage influx was similar between WT and DAP12 KO mice, despite differences in renal MCP1 level making the latter hypothesis less likely. The exact reason for the delayed macrophage influx remains speculative. The alterations in inflammation in TREM1/3 KO and DAP12 KO mice did not affect development of fibrosis given that myofibroblast accumulation and collagen deposition increased similarly in all three groups (figure 5). In addition, tubular apoptosis and renal damage increased similarly in all three groups during progression of UUO. However, early after UUO, tubular damage was slightly higher in TREM1/3 KO mice (day 1) and DAP12 KO mice (day 1+3) which was in line with more edema in these mice (figure 6). Enhanced tubular damage upon UUO was also observed in TLR4 KO mice [12], possibly because of TLR4 involvement in regulation of adhesion molecules by fibroblasts, epi- and endothelial cells and its role in tubular architecture and integrity [39]–[41]. TREM1 has been described to interact with TLR4 [42] [24] and we showed TREM1 expression on murine TECs. Possibly, similar mechanisms may play part in enhanced tubular damage in TREM1/3 KO mice as seen in TLR4 KO mice but this remains to be investigated in future.

During UUO, TREM1/3 KO mice displayed only partial similar phenotype compared to DAP12 KO mice. This implicates that other DAP12-related receptors might be involved, for instance Sirpb1, Pilrb, Cd200r3+4, Clec5a, TREM2, Cd300lb, AF251705 (MAIR-II), Siglec-h, and Siglec15 (reviewed in [25]). In a screening for these DAP12 associated myeloid receptors upon UUO, we observed increased mRNA levels of all mentioned genes (figure 7). Earlier studies have shown that Cd300lb[43], Pilrb[44], Clec5a[45] [46], MAIR-II[47], Siglec-h[48], Siglec-15[49] are involved in induction of inflammation. So far, only the contribution of DAP12-associated myeloid receptors Cd300lb (commonly described as LMIR5) has been investigated in renal diseases showing that LMIR5 deficiency ameliorates mouse kidney IRI [50]. LMIR5 deficient mice displayed less renal inflammatory mediators, including MCP1, neutrophil influx and renal damage following IR [50]. If LMIR-5, or other DAP12-associted receptors except for TREM1/3, play part in UUO remains to be investigated and is beyond the scope of this paper. In conclusion, our results show that DAP12, partly through TREM1/3, is involved in renal inflammation during progression of UUO whereas TREM1-DAP12 does not play part in renal fibrosis.

## Supporting Information

Figure S1 **TREM1 protein expression on TEC.** Primary TECs were isolated, cultured and TREM1 protein expression was determined by western blot. TECs from WT mice were stimulated with 1 or 5 ng TGF-β1/ml medium for 3 days. Cell lysates were obtained and quantified for TREM1 protein expression (A). Densometric quantification analysis of western blot is displayed in B, control was set to 1. Data are mean ± SD, N = 2 per group. AU = arbitrary units.
(TIF)
