# Supplementary figures and images for: Role of TREM1-DAP12 in Renal Inflammation during Obstructive Nephropathy
Source: PLoS One. 2013 Dec 16;8(12):e82498. doi: 10.1371/journal.pone.0082498 (PMC3864959; doi:10.1371/journal.pone.0082498)

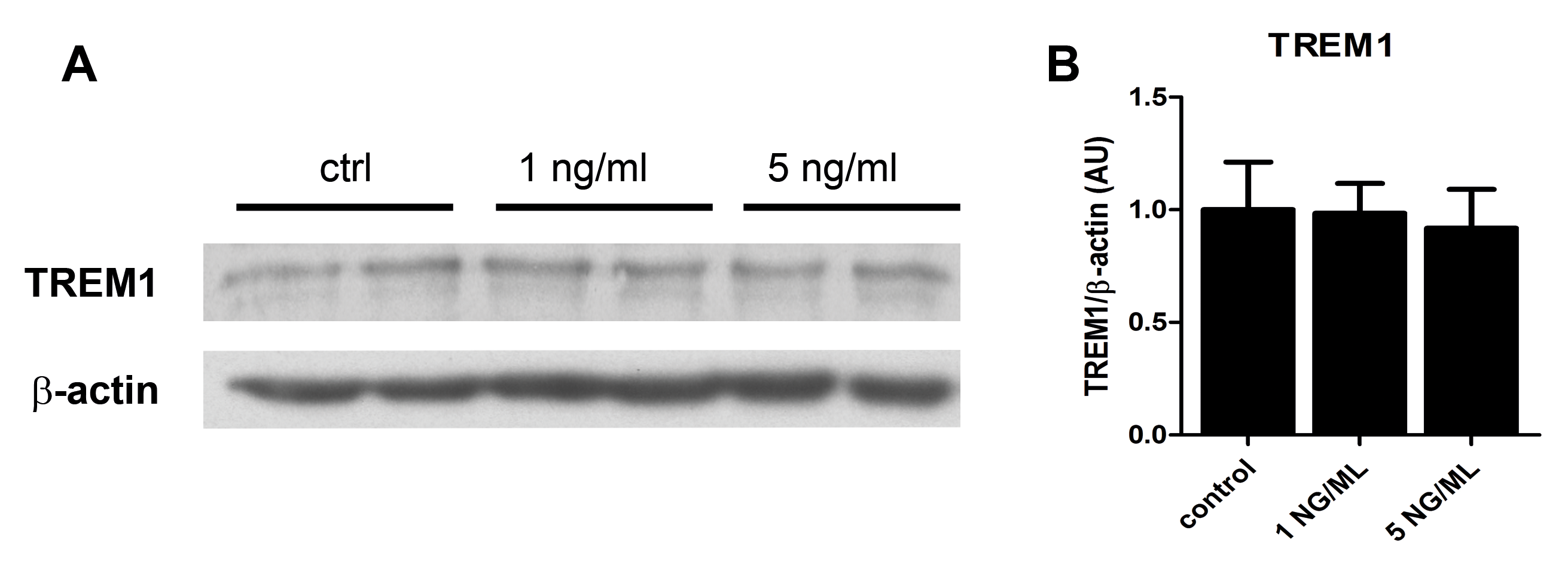

Supplement: Figure S1 — TREM1 protein expression on TEC. Primary TECs were isolated, cultured and TREM1 protein expression was determined by western blot. TECs from WT mice were stimulated with 1 or 5 ng TGF-β1/ml medium for 3 days. Cell lysates were obtained and quantified for TREM1 protein expression (A). Densometric quantification analysis of western blot is displayed in B, control was set to 1. Data are mean ± SD, N = 2 per group. AU = arbitrary units. (TIF) [file pone.0082498.s001.tif]
